# Zoonotic Pathogens in Wildlife Traded in Markets for Human Consumption, Laos

**DOI:** 10.3201/eid2804.210249

**Published:** 2022-04

**Authors:** Pruksa Nawtaisong, Matthew T. Robinson, Khongsy Khammavong, Phonesavanh Milavong, Audrey Rachlin, Sabine Dittrich, Audrey Dubot-Pérès, Malavanh Vongsouvath, Paul F. Horwood, Philippe Dussart, Watthana Theppangna, Bounlom Douangngeum, Amanda E. Fine, Mathieu Pruvot, Paul N. Newton

**Affiliations:** Lao-Oxford-Mahosot Hospital-Wellcome Trust Research Unit, Mahosot Hospital, Vientiane, Laos (P. Nawtaisong, M.T. Robinson, A. Rachlin, S. Dittrich, A. Dubot-Pérès, M. Vongsouvath, P.N. Newton);; University of Oxford Centre for Tropical Medicine and Global Health, Oxford, UK (M.T. Robinson, S. Dittrich, A. Dubot-Pérès, P.N. Newton);; Wildlife Conservation Society, Bronx, New York, USA (K. Khammavong, P. Milavong, A.E. Fine, M. Pruvot); Unité des Virus Émergents (UVE) Aix-Marseille Univ-IRD 190-Inserm 1207),; Marseille, France (A. Dubot-Pérès); Institut Pasteur du Cambodge, Phnom Penh, Cambodia (P.F. Horwood, P. Dussart);; National Animal Health Laboratory, Ministry of Agriculture, Vientiane (W. Theppangna, B. Douangngeum);; University of Calgary, Calgary, Alberta, Canada (M. Pruvot)

**Keywords:** zoonoses, Laos, *Rickettsia*, *Leptospira*, *Orientia tsutsugamushi*, wildlife, illegal trade, bushmeat, bacteria

## Abstract

We tested animals from wildlife trade sites in Laos for the presence of zoonotic pathogens. *Leptospira* spp. were the most frequently detected infectious agents, found in 20.1% of animals. *Rickettsia typhi* and *R. felis* were also detected. These findings suggest a substantial risk for exposure through handling and consumption of wild animal meat.

Consumption of wildlife meat drives emerging infectious diseases (*1*), often amplified by human encroachment into natural areas and changes in land use. Wildlife trade and consumption have been responsible for outbreaks of diseases such as HIV-1 (*2*), Ebola (*3*), and monkeypox (*4*) and possibly for the coronavirus disease pandemic (*5*). Wildlife markets bring diverse species into contact, usually in dense and unsanitary conditions, enabling mixing, amplification, and transmission of pathogens among species, including humans (*6*). Small mammals host diverse pathogenic bacteria and viruses (*7*), but little investigation of endemic bacteria transmission has occurred. Determining pathogens present in traded wildlife is vital to guide appropriate measures to combat zoonotic diseases and document societal and environmental costs of wildlife trade.

## The Study

During December 2014–September 2017, we collected samples from 9 wildlife trade hotspots (*8*) and 2 roadside stalls (hereafter all referred to as trade sites) in Laos (Figure; Appendix Table 1). In addition, 3 Provincial Offices of Forest Inspection (POFI) collected samples from wildlife confiscated in markets by law enforcement. After identifying wildlife at trade sites (*9*), we asked vendors for permission to sample their animals. Depending on whether the animal was alive, dead, or butchered, we collected urogenital swabs, urine and blood samples, and kidney, liver, and spleen tissue samples (Appendix Table 2). 

**Figure:**
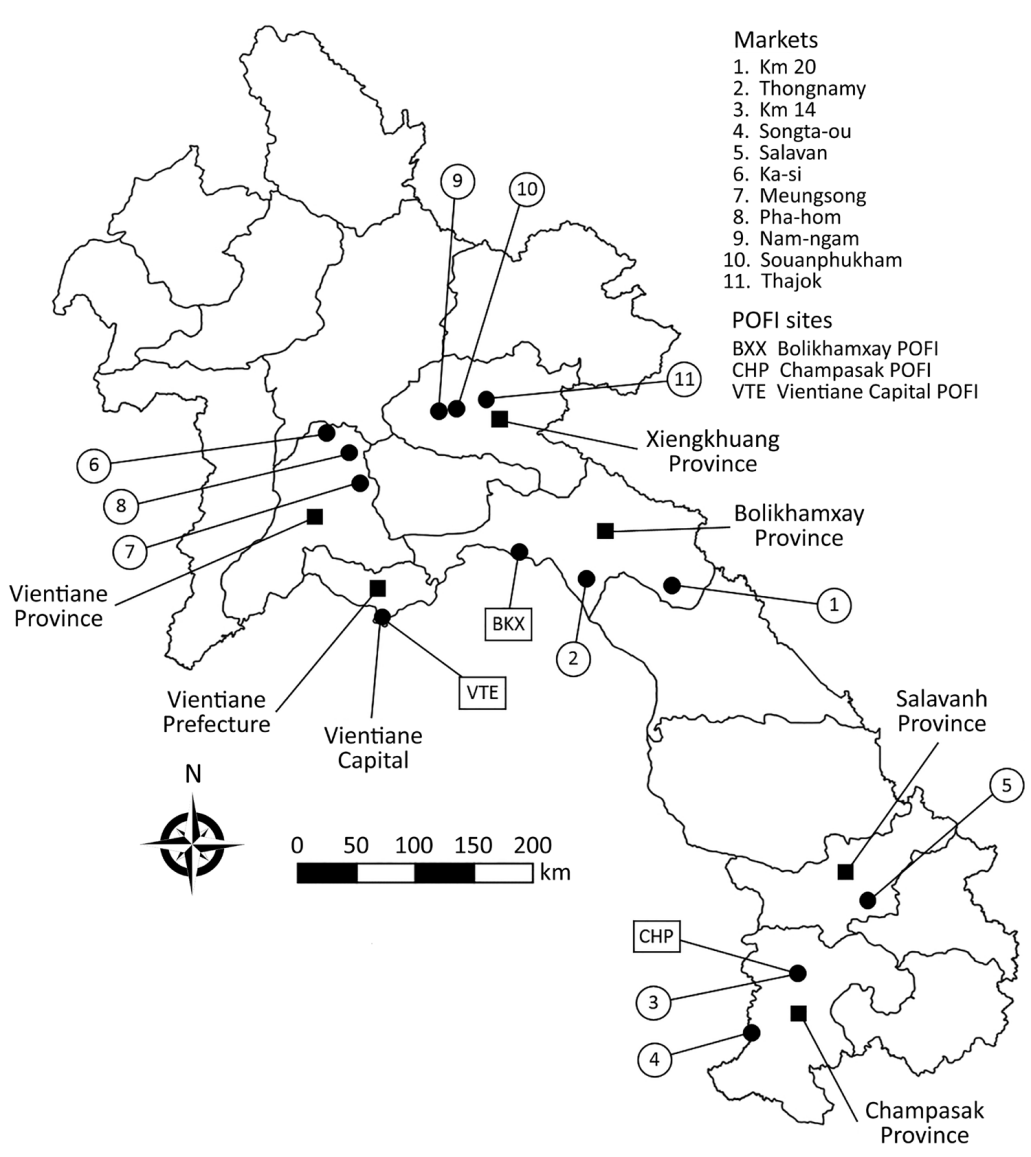
Wildlife trade sites and POFI sites (black circles) where wildlife samples were collected for study of zoonotic pathogens in wildlife traded in markets for human consumption, Laos. Provinces are labeled with black squares. POFI, Provincial Office of Forestry Inspection.

We extracted nucleic acid using QIAamp Viral RNA Mini Kits (QIAGEN, https://www.qiagen.com) with modifications (Appendix). We conducted PCRs targeting *Leptospira* spp., *Rickettsia* spp., *Orientia tsutsugamushi*, Anaplasmataceae, *Ehrlichia chaffeensis*, *Anaplasma phagocytophilum*, *Coxiella burnetti*, flaviviruses, hantavirus, dengue virus, Zika virus, and universal bacterial 16S rRNA (Appendix Table 3). Where necessary, PCR products were sequenced (Macrogen Inc., https://www.macrogen.com) and compared against GenBank through blastn (https://blast.ncbi.nlm.nih.gov). We performed descriptive, univariate, and multivariate analyses by using R version 3.6.2 (https://www.r-project.org). We assessed the effect of the wild meat processing status (alive, fresh, or frozen) on the risk for *Leptospira* detection by using a mixed effects logistic regression with species as random effect. Statistical significance was set at α = 0.05 (Appendix).

We collected 717 samples from 359 animals (trade sites: 461 samples from 324 animals; POFI: 256 samples from 35 animals); animals sampled were from >37 identifiable vertebrate species from 12 families (Appendix Table 4). Most were Sciuridae squirrels (73.0%, 262/359) and represented 16 species, most frequently Pallas’s squirrel (*Callosciurus erythraeus*) (20.3%, 73/359). From trade sites, 69 animals (21.3%, 95% CI 17.0%–26.2%) had >1 samples positive for >1 pathogens in 10 of 11 sites (90.9%, 95% CI 57.1%–99.5%) (Appendix Table 5). Of 324 animals tested, 65 (20.1%, 95% CI 15.9%–24.9%) were positive for *Leptospira* spp.; 4/41 were positive for *Rickettsia* spp. (9.8%, 95% CI 3.2%–24.1%), 0 for *O. tsutsugamushi* (0%, 95% CI 0%–10.7%), and 2 for Anaplasmataceae (4.9%, 95% CI 0.8%–17.8%) (Table 1). Positivity was higher among animals collected by POFI; 25/35 (71.4%) animals tested positive for >1 pathogens. Of those, 9 were positive for *Leptospira* spp. (25.7%, 95% CI 13.1%–43.6%), 20 for *Rickettsia* spp. (57.1%, 95% CI 39.5%–73.2%), 2 for *O. tsutsugamushi* (5.7%, 95% CI 1.0%–20.5%), and 6 for Anaplasmataceae (17.1%, 95% CI 7.2%–34.3%) (Table 2). Sequencing identified *R. typhi*, *R. felis*, *R. conorii*, an *Anaplasma* species (either *A. centrale*, *A. capra*, or *A. marginale*), *A. platys*, *A. bovis*, *A. phagocytophilum*, *Ehrlichia chaffeensis*, *Lactococcus garvieae*, and *Kurthia populi* (Tables 1, 2). No samples were positive for *C. burnetii* (0/76), flaviviruses (0/359), dengue virus (0/359), or Zika virus (0/358).

**Table 1:** Zoonotic pathogens detected and animal species and sample types that tested positive in wildlife collected from trade sites, Laos*

Organism	No. positive/no. tested				Sequencing identity match, %†
	Animals	Species	Samples	Sample types	
*Leptospira* spp.	65/324	*Callosciurus finlaysonii* squirrel, 13/28	72/461	URO, 58/312	NA
		*C. erythraeus* squirrel, 8/56		SPL, 1/3	
		*Paradoxurus hermaphroditus* civet, 10/22		KID, 2/6	
		*C. inornatus* squirrel, 7/34		LIV, 1/40	
		*Dremomys rufigenis* squirrel, 5/35		BLD, 9/85	
		*Menetes berdmorei* ground squirrel, 4/29		URI, 1/15	
		*Rhizomys pruinosus* rat, 3/21			
		*Arctogalidia trivirgata* civet, 2/2			
		*Petaurista philippensis* flying squirrel, 1/9			
		*Atherurus macrourus* porcupine, 1/1			
		*Belomys pearsonii* flying squirrel, 1/12			
		*Eonycteris spelaea* bat, 1/3			
		*Hylopetes alboniger* flying squirrel, 1/5			
		*H. phayrei* flying squirrel, 1/9			
		*H. spadiceus* flying squirrel, 1/2			
		*Muntiacus muntjak* deer, 1/1			
		*Paguma larvata* civet, 1/2			
		*Prionailurus bengalensis* cat, 1/3			
		Rhizomys *sumatrensis* rat, 1/6			
		*Tupaia belangeri* treeshrew, 1/3			
		Unknown Sciuridae squirrel, 1/2			
*Rickettsia* spp.	1/41	*P. philippensis* flying squirrel, 1/2	1/68	LIV, 1/40	NA
*Rickettsia felis*†	2/41	*D. rufigenis* squirrel, 1/11	2/68	LIV, 2/40	98–100
		*P. hermaphroditus* civet, 1/6			
*R*. *typhi*†	1/41	*D. rufigenis* squirrel, 1/11	1/68	LIV, 1/40	93
*Anaplasma platys*†	1/41	*P. hermaphroditus* civet, 1/6	1/68	KID, 1/6	98
*A*. *centrale*	1/41	*M. muntjak* deer, 1/1	5/68	KID, 1/6	98.8–99.6 (*A. centrale*)
*A. capra*				LIV, 3/40	98.8–99.6 (*A. capra*)
*A. marginale*†				SPL, 1/3	98.8 (*A. marginale*)

*BLD, blood; KID, kidney; LIV, liver; NA, not applicable; SPL, spleen; URI, urine; URO, urogenital swab. †Organism identified by sequencing of PCR products and identity match given in the right-hand column. All nucleotide sequences were submitted to GenBank under accession nos. MW407963–MW407984 and MW411434–MW411439.

**Table 2:** Zoonotic pathogens detected and animal species and sample types that tested positive in wildlife collected from POFI sites*

Organism	No. positive/no. tested				Sequencing identity match, %†
	Animals	Species	Samples	Sample types	
*Leptospira* spp.	9/35	*Callosciurus finlaysonii* squirrel, 1/1	46/256	SPL, 17/69	NA
		*Callosciurus erythraeus* squirrel, 4/17		KID, 14/91	
		*Callosciurus inornatus* squirrel, 2/6		LIV, 14/92	
		*Petaurista philippensis* flying squirrel, 1/5		BLD, 1/3	
		*Catopuma temminckii* cat, 1/1			
*Orientia tsutsugamushi*	2/34	*C. erythraeus* squirrel, 2/17	2/252	SPL, 2/252	NA
*Rickettsia* spp.	12/35	*C. erythraeus* squirrel, 5/17	70/252	LIV, 30/92	NA
		*P. philippensis* flying squirrel, 2/5		KID, 25/91	
		*C. inornatus* squirrel, 2/6		SPL, 15/69	
		*Paradoxurus hermaphroditus* civet, 1/2			
		*Catopuma temminckii* cat, 1/1			
		*Ratufa bicolor* squirrel, 1/1			
*Rickettsia conorii*†	1/35	*P. philippensis* flying squirrel, 1/5	1/252	LIV, 1/92	99
*R. felis*†	1/35	*C. erythraeus* squirrel, 1/17	2/252	LIV, 1/92	98
				SPL, 1/69	
*R. typhi*	6/35	*C. erythraeus* squirrel, 6/17	7/252	KID, 4/91	NA
				LIV, 2/92	
				SPL, 1/69	
Anaplasmataceae	1/34	*C. erythraeus* squirrel, 1/17	3/252	KID, 2/91	NA
				SPL, 1/69	
*Anaplasma bovis*†	1/34	*C. erythraeus* squirrel, 1/17	7/252	KID, 1/91	99.7–100
				LIV, 3/92	
				SPL, 3/69	
*A. phagocytophilum*†	2/34	*Catopuma temminckii* cat, 1/1	4/252	KID, 2/91	98–99
		*P. philippensis* flying squirrel, 1/4		SPL, 2/69	
*Ehrlichia* spp*./E. chaffeensis*†	1/34	Unknown Muridae rat, 1/1	1/252	SPL, 1/69	97 (*Ehrlichia* spp.)
					97 (*E. chaffeensis*)
*Kurthia populi*†	1/34	*C. erythraeus* squirrel, 1/17	1/252	LIV, 1/92	98
*Lactococcus garvieae*†	1/34	*C. erythraeus* squirrel, 1/17	1/252	SPL, 1/69	99

*BLD, blood; KID, kidney; LIV, liver; NA, not applicable; POFI, Provincial Office of Forestry Inspection; SPL, spleen; URI, urine; URO, urogenital swab. †Organism identified by sequencing of PCR products and identity match given in righthand column. All nucleotide sequences were submitted to GenBank under accession nos. MW407963–MW407984 and MW411434–MW411439.

Among species for which >10 individual animals were sampled in trade sites, 2 had particularly high proportions of *Leptospira* spp.–positive specimens: the variable squirrel (*Callosciurus finlaysonii*) (13/28; 46.4% 95% CI 28.0%–65.8%) and the common palm civet (*Paradoxurus hermaphroditus)* (10/22; 45.5%, 95% CI 25.2%–67.3%). *Leptospira* spp.–positivity was higher in dry (50/195; 25.6%, 95% CI 19.8%–32.5%) than wet season (15/129; 11.6%, 95% CI 6.9%–18.8%) (χ^2^ = 8.7; p = 0.003). Data disaggregation by species and province suggested that observed seasonality was driven by results in common palm civets and variable squirrels in Champasak Province. No association was detected between the probability of an animal testing positive for *Leptospira* and the animal being alive (3/22; 14%, 95% CI 3.6%–36%), freshly dead (58/293; 20%, 95% CI 16%–25%; p = 0.6), or frozen (4/9; 44%, 95% CI 15%–77%; p = 0.1). In a subset of *Leptospira* spp.–positive animals with multiple samples, 75% (18/24; 95% CI 53%–89%) of urogenital swab samples and 50% (9/18; 95% CI 29%–71%) of blood samples were positive (p = 0.11 by Fisher exact test). *Rickettsia* spp. were detected exclusively in solid organs (liver, kidney, and spleen).

Zoonotic pathogens were nearly ubiquitous across sites; 10/11 sites yielded >1 pathogens. Squirrels are frequently traded in Lao markets (*8*) and had the greatest pathogen diversity in this study. *Leptospira* spp. was identified most frequently, found in 20.1% of animals (>45% in variable squirrels and common palm civets). Variable squirrels are commonly traded, often in batches of 2 to 3 squirrels (*8*); hence, on average, someone purchasing 3 variable squirrels would have an 83% likelihood of buying >1 infected squirrel (p = 1 – (1 – prevalence)^3^ = 1 – 0.55^3^ = 0.83). The higher risk for *Leptospira* detection in the dry season is at odds with the typically described correlation of transmission with precipitation and flooding (*10*), suggesting that much remains to be understood of *Leptospira* ecology. Other studies have shown higher prevalence in rats (*11*), and although we are confident of the results from trade sites, storage of animals from POFI sites might have resulted in cross-contamination, which warrants cautious interpretation of results in this subset. Among *Leptospira* spp.-positive animals, detection was more likely in urogenital swab samples, highlighting the risk for transmission through infected urine (*10*). Although reservoir rodents are characterized by chronic renal infections, septicemia occurs during initial infection (*10*), and the high proportion of positive blood samples indicates a public health risk in relation to the consumption of uncooked or undercooked meat, organs, and blood. The PCR used to detect leptospires is specific for pathogenic and intermediate species (Appendix Table 3), but we could not confirm their human pathogenicity. The high volume of squirrel trade combined with high infection frequency suggests a high risk for exposure among wildlife consumers. Because leptospirosis is a key cause of fever in rural Laos (*12*), further work is needed to learn more about the relevance of contact with wildlife through trade and consumption.

The Rickettsiales species identified here are known to cause human infections in Laos (*13*). *R. typhi* causes murine typhus, a major underrecognized cause of fever (*13*). *O. tsutsugamushi* is responsible for up to 23% of fever (*14*), and although commonly associated with ground-dwelling rodents, the vectors (*Leptotrombidium* mites) parasitize squirrels (*15*), and *O. tsutsugamushi* has been isolated from *Callosciurus notatus* squirrels in Malaysia (*16*). Other bacteria identified are reviewed elsewhere (Appendix Table 6).

Although many of the human pathogens identified are transmitted by arthropod vectors, we found few arthropods in the wildlife sampled, probably because vectors leave animals quickly after animal death (*17*). Therefore, because most market vendors sell dead animals obtained from hunters or intermediaries (*8*), vendors are less likely to be exposed to disease vectors, and hunters are possibly at greater risk than market vendors or consumers. *O. tsutsugamushi* and *R. typhi* can cause infections through aerosol exposure, bites from infected animals, and needlestick injuries (*18*), but whether such routes of infection occur at trade sites is unclear. The frequent occurrence of *Leptospira*, which can be transmitted by direct contact with abraded skin and mucous membranes, may pose health risks to hunters, vendors, and consumers.

Appendix Additional information about zoonotic pathogens in wildlife traded in markets for human consumption, Laos.

## Footnotes

3 Current affiliation: Campus Biotech, Geneva, Switzerland; University of Oxford Centre for Tropical Medicine and Global Health, Oxford, UK.
