# Supplementary material for: Zoonotic Pathogens in Wildlife Traded in Markets for Human Consumption, Laos
Source: Emerg Infect Dis. 2022 Apr;28(4):860–4. doi: 10.3201/eid2804.210249 (PMC8962878; doi:10.3201/eid2804.210249)
Supplement: Appendix — Additional information about zoonotic pathogens in wildlife traded in markets for human consumption, Laos. [file 21-0249-Techapp-s1.pdf]

Article DOI: https://doi.org/10.3201/eid2804.210249

Zoonotic Pathogens in Wildlife Traded in Markets for Human Consumption, Laos

Appendix

Supplementary Methods

Data Collection

Identification of animals was done to species level when possible by using an established regional field guide (*1*). Basic information, such as the status of the animal (live or dead) and storage status (frozen or fresh), was recorded.

Extraction of Nucleic Acids

We extracted samples by using the QIAamp Viral RNA Mini Kits (QIAGEN, https://www.qiagen.com). The manufacturer's protocol was modified following manufacturer's recommendations (*2*) and DNA and RNA were extracted simultaneously. Urogenital swabs were first centrifuged to release cells from the swabs into the supernatant, which was then transferred into new tubes. For blood and urine, 200 µL of sample was used. Buffer AL (200 µL) and Proteinase K (20 µL) were added to the samples and incubated at 56°C for 30 minutes. For liver, spleen, and kidney tissue, 0.025 g of tissue was transferred to a tube containing glass beads. Buffer ATL (200 µL) and Proteinase K (20 µL) were added and the sample vortexed for 30 minutes, then incubated as described previously. To check for the presence of PCR inhibitors, 10 µL of T4/MS2 phage solution (*3*) was added after cell lysis. The extraction process was continued as per manufacturer's protocol with minor adjustments: 800 µL AVL/carrier RNA buffer, 200 µL ethanol, and 500 µL AW1 and AW2. Total nucleic acid was eluted in 100 µL Buffer AVE.

Statistical Analysis

Descriptive, univariate, and multivariate analyses were done using R version 3.6.2 (https://www.r-project.org). Confidence interval around prevalence estimates and other proportions used binomial confidence intervals. The effect of season on *Leptospira* prevalence

was initially assessed by a χ^2 test. To further test the season effect in different provinces, we used a logistic regression with season, province, and their interaction as explanatory variables. The effect of the wild meat preservation method on the risk for *Leptospira* detection was assessed by using a mixed effects logistic regression with species as random effect, using the R package *lme4*. In a subset of *Leptospira* spp.-positive animals for which multiple sample types were available for testing (i.e., animals have multiple sample types, and $\geq$1 of them is positive for *Leptospira*), we compared the proportion of positive results in genital swab samples and blood samples by using a Fisher exact test.

Finally, to further demonstrate the significance of the high prevalence of *Leptospira* spp. found in squirrels, we estimated the probability of a consumer being exposed to *Leptospira* in the simple and common scenario where a consumer purchases 3 variable squirrels. With a known prevalence *p* of *Leptospira* spp. in variable squirrels, the probability of a consumer to purchase $\geq$1 infected animal is one minus the probability to purchase no infected animals. Assuming that the infection status of individual squirrels on a stall is independent, this is P(purchasing $\geq$1 infected squirrel among) = $1-(1-p)^3$.

References

1. Francis CM. A field guide to the mammals of southeast Asia. London: New Holland; 2008.

2. QIAGEN. QIAamp DNA mini and blood mini handbook. 5th ed. 2016. pp 72.

3. Ninove L, Nougairede A, Gazin C, Thirion L, Delogu I, Zandotti C, et al. RNA and DNA bacteriophages as molecular diagnosis controls in clinical virology: a comprehensive study of more than 45,000 routine PCR tests. PLoS One. 2011;6:e16142. PubMed https://doi.org/10.1371/journal.pone.0016142

4. Jiang J, Chan TC, Temenak JJ, Dasch GA, Ching WM, Richards AL. Development of a quantitative real-time polymerase chain reaction assay specific for *Orientia tsutsugamushi*. Am J Trop Med Hyg. 2004;70:351–6. PubMed https://doi.org/10.4269/ajtmh.2004.70.351

5. Jiang J, Stromdahl EY, Richards AL. Detection of *Rickettsia parkeri* and *Candidatus* Rickettsia andeanae in *Amblyomma maculatum* Gulf Coast ticks collected from humans in the United States. Vector Borne Zoonotic Dis. 2012;12:175–82. PubMed https://doi.org/10.1089/vbz.2011.0614

6. Slack A, Symonds M, Dohnt M, Harris C, Brookes D, Smythe L. Evaluation of a modified Taqman assay detecting pathogenic *Leptospira* spp. against culture and *Leptospira*-specific IgM enzyme-linked immunosorbent assay in a clinical environment. Diagn Microbiol Infect Dis. 2007;57:361–6. PubMed https://doi.org/10.1016/j.diagmicrobio.2006.10.004

7. Smythe LD, Smith IL, Smith GA, Dohnt MF, Symonds ML, Barnett LJ, et al. A quantitative PCR (TaqMan) assay for pathogenic *Leptospira* spp. BMC Infect Dis. 2002;2:13. PubMed https://doi.org/10.1186/1471-2334-2-13

8. Thaipadungpanit J, Chierakul W, Wuthiekanun V, Limmathurotsakul D, Amornchai P, Boonslip S, et al. Diagnostic accuracy of real-time PCR assays targeting 16S rRNA and lipL32 genes for human leptospirosis in Thailand: a case-control study [Erratum in: PLoS One. 2011;6]. PLoS One. 2011;6:e16236. PubMed https://doi.org/10.1371/journal.pone.0016236

9. Parola P, Roux V, Camicas JL, Baradji I, Brouqui P, Raoult D. Detection of ehrlichiae in African ticks by polymerase chain reaction. Trans R Soc Trop Med Hyg. 2000;94:707–8. PubMed https://doi.org/10.1016/S0035-9203(00)90243-8

10. Loftis AD, Massung RF, Levin ML. Quantitative real-time PCR assay for detection of *Ehrlichia chaffeensis.* J Clin Microbiol. 2003;41:3870–2. PubMed https://doi.org/10.1128/JCM.41.8.3870-3872.2003

11. Courtney JW, Kostelnik LM, Zeidner NS, Massung RF. Multiplex real-time PCR for detection of *anaplasma phagocytophilum* and *Borrelia burgdorferi.* J Clin Microbiol. 2004;42:3164–8. PubMed https://doi.org/10.1128/JCM.42.7.3164-3168.2004

12. Tilburg JJ, Horrevorts AM, Peeters MF, Klaassen CH, Rossen JW. Identification by genotyping of a commercial antigen preparation as the source of a laboratory contamination with *Coxiella burnetii* and as an unexpected rich source of control DNA. J Clin Microbiol. 2011;49:383–4. PubMed https://doi.org/10.1128/JCM.01491-10

13. Moureau G, Temmam S, Gonzalez JP, Charrel RN, Grard G, de Lamballerie X. A real-time RT-PCR method for the universal detection and identification of flaviviruses. Vector Borne Zoonotic Dis. 2007;7:467–77. PubMed https://doi.org/10.1089/vbz.2007.0206

14. Leparc-Goffart I, Baragatti M, Temmam S, Tuiskunen A, Moureau G, Charrel R, et al. Development and validation of real-time one-step reverse transcription-PCR for the detection and typing of dengue viruses. J Clin Virol. 2009;45:61–6. PubMed https://doi.org/10.1016/j.jcv.2009.02.010

15. Mohamed N, Nilsson E, Johansson P, Klingström J, Evander M, Ahlm C, et al. Development and evaluation of a broad reacting SYBR-green based quantitative real-time PCR for the detection of different hantaviruses. J Clin Virol. 2013;56:280–5. PubMed https://doi.org/10.1016/j.jcv.2012.12.001

16. Edwards U, Rogall T, Blöcker H, Emde M, Böttger EC. Isolation and direct complete nucleotide determination of entire genes. Characterization of a gene coding for 16S ribosomal RNA. Nucleic Acids Res. 1989;17:7843–53. PubMed https://doi.org/10.1093/nar/17.19.7843

17. Cleenwerck I, Camu N, Engelbeen K, De Winter T, Vandemeulebroecke K, De Vos P, et al. *Acetobacter ghanensis* sp. nov., a novel acetic acid bacterium isolated from traditional heap fermentations of Ghanaian cocoa beans. Int J Syst Evol Microbiol. 2007;57:1647–52. PubMed https://doi.org/10.1099/ijs.0.64840-0

18. Ninove L, Nougairede A, Gazin C, Thirion L, Delogu I, Zandotti C, et al. RNA and DNA bacteriophages as molecular diagnosis controls in clinical virology: a comprehensive study of more than 45,000 routine PCR tests. PLoS One. 2011;6:e16142. PubMed https://doi.org/10.1371/journal.pone.0016142

19. Phongmany S, Rolain J-M, Phetsouvanh R, Blacksell SD, Soukkhaseum V, Rasachack B, et al. Rickettsial infections and fever, Vientiane, Laos. Emerg Infect Dis. 2006;12:256–62. PubMed https://doi.org/10.3201/eid1202.050900

20. Angelakis E, Mediannikov O, Parola P, Raoult D. *Rickettsia felis*: the complex journey of an emergent human pathogen. Trends Parasitol. 2016;32:554–64. PubMed https://doi.org/10.1016/j.pt.2016.04.009

21. Kurup A, Issac A, Loh JP, Lee TB, Chua R, Bist P, et al. Scrub typhus with sepsis and acute respiratory distress syndrome. J Clin Microbiol. 2013;51:2787–90. PubMed https://doi.org/10.1128/JCM.00463-13

22. Choe S, Lee D, Park H, Jeon HK, Lee Y, Na KJ, et al. *Catenotaenia dendritica* (Cestoda: Catenotaeniidae) and three ectoparasite species in the red squirrel, *Sciurus vulgaris*, from Cheongju, Korea. Korean J Parasitol. 2016;54:509–18. PubMed https://doi.org/10.3347/kjp.2016.54.4.509

23. Walker JS, Gan E, Muul I, Muul I; Chan Teik Chye. Involvement of small mammals in the transmission of scrub typhus in Malaysia: isolation and serological evidence. Trans R Soc Trop Med Hyg. 1973;67:838–45. PubMed https://doi.org/10.1016/0035-9203(73)90012-6

24. Atif FA. *Anaplasma marginale* and *Anaplasma phagocytophilum*: Rickettsiales pathogens of veterinary and public health significance. Parasitol Res. 2015;114:3941–57. PubMed https://doi.org/10.1007/s00436-015-4698-2

25. Breitschwerdt EB, Hegarty BC, Qurollo BA, Saito TB, Maggi RG, Blanton LS, et al. Intravascular persistence of *Anaplasma platys, Ehrlichia chaffeensis*, and *Ehrlichia ewingii* DNA in the blood of a dog and two family members. Parasit Vectors. 2014;7:298. PubMed https://doi.org/10.1186/1756-3305-7-298

26. Li H, Zheng Y-C, Ma L, Jia N, Jiang B-G, Jiang R-R, et al. Human infection with a novel tick-borne *Anaplasma* species in China: a surveillance study. Lancet Infect Dis. 2015;15:663–70. PubMed https://doi.org/10.1016/S1473-3099(15)70051-4

27. Yang J, Liu Z, Niu Q, Mukhtar MU, Guan G, Liu G, et al. A novel genotype of "*Anaplasma capra*" in wildlife and its phylogenetic relationship with the human genotypes. Emerg Microbes Infect. 2018;7:210. PubMed https://doi.org/10.1038/s41426-018-0212-0

28. Costa SC, de Magalhães VC, de Oliveira UV, Carvalho FS, de Almeida CP, Machado RZ, et al. Transplacental transmission of bovine tick-borne pathogens: Frequency, co-infections and fatal neonatal anaplasmosis in a region of enzootic stability in the northeast of Brazil. Ticks Tick Borne Dis. 2016;7:270–5. PubMed https://doi.org/10.1016/j.ttbdis.2015.11.001

29. Howden KJ, Geale DW, Paré J, Golsteyn-Thomas EJ, Gajadhar AA. An update on bovine anaplasmosis (*Anaplasma marginale*) in Canada. Can Vet J. 2010;51:837–40. PubMed

30. Dong T, Qu Z, Zhang L. Detection of *A. phagocytophilum* and *E. chaffeensis* in patient and mouse blood and ticks by a duplex real-time PCR assay. PLoS One. 2013;8:e74796. PubMed https://doi.org/10.1371/journal.pone.0074796

31. Chan JF, Woo PC, Teng JL, Lau SK, Leung SS, Tam FC, et al. Primary infective spondylodiscitis caused by *Lactococcus garvieae* and a review of human *L. garvieae* infections. Infection. 2011;39:259–64. PubMed https://doi.org/10.1007/s15010-011-0094-8

32. Ongrádi J, Stercz B, Kövesdi V, Nagy K, Chatlynne L. Isolation of *Kurthia gibsonii* from non-gonorrheal urethritis: implications for the pathomechanism upon surveying the literature. Acta Microbiol Immunol Hung. 2014;61:79–87. PubMed https://doi.org/10.1556/amicr.61.2014.1.8

Appendix Table 1. Location and date of wildlife sampled from markets, Laos*

ID	Province	Site	Site classification	Season	Date of visit	No. animals sampled	No. animals sampled (% of total)	No. animals positive by PCR (% of animals from site)
1	Bolikhamxay	Km 20 market	Trade	Dry	2016 Jan 22	8	49 (13.7)	5 (10.2)
				Dry	2016 Jan 23	3		
				Dry	2016 Jan 24	6		
				Dry	2016 Feb 26	3		
				Dry	2016 Feb 27	6		
				Dry	2016 Feb 28	10		
				Wet	2015 Aug 20	6		
				Wet	2015 Aug 21	5		
				Wet	2017 Sep 19	2		
2	Bolikhamxay	Thongnamy	Trade	Dry	2016 Jan 25	9	23 (6.4)	8 (34.8)
				Dry	2016 Jan 26	4		
				Dry	2016 Mar 1	3		
				Wet	2016 May 4	2		
				Wet	2016 May 5	5		
3	Champasak	Km 14 market	Trade	Dry	2015 Dec 17	4	49 (13.7)	15 (30.6)
				Dry	2015 Dec 18	5		
				Dry	2015 Dec 19	5		
				Dry	2016 Feb 5	5		
				Dry	2016 Feb 6	1		
				Dry	2016 Feb 7	6		
				Dry	2016 Feb 9	7		
				Wet	2015 May 23	7		
				Wet	2015 May 24	4		
				Wet	2015 May 25	4		
				Wet	2015 May 26	1		
4	Champasak	Songta-ou	Trade	Dry	2015 Nov 2	1	55 (15.3)	12 (21.8)
				Dry	2015 Dec 16	11		
				Dry	2015 Dec 21	8		
				Dry	2016 Feb 8	10		
				Dry	2016 Feb 10	9		
				Wet	2015 May 20	9		
				Wet	2015 May 22	7		
5	Saravanh	Salavan Market	Trade	Dry	2015 Dec 23	5	44 (12.3)	9 (20.5)
				Dry	2015 Dec 24	4		
				Dry	2015 Dec 25	2		
				Dry	2016 Feb 11	6		
				Dry	2016 Feb 12	2		
				Dry	2016 Feb 13	3		
				Dry	2016 Feb 14	3		
				Dry	2016 Apr 30	5		
				Wet	2015 May 28	7		
				Wet	2015 May 29	4		
				Wet	2016 May 1	3		
6	Vientiane	Ka-si	Trade	Dry	2016 Mar 15	10	40 (11.1)	6 (15.0)
				Dry	2016 Mar 16	6		
				Wet	2015 Aug 8	5		

ID	Province	Site	Site classification	Season	Date of visit	No. animals sampled	No. animals sampled (% of total)	No. animals positive by PCR (% of animals from site)
				Wet	2015 Aug 9	2		
				Wet	2015 Sep 19	8		
				Wet	2015 Sep 20	5		
				Wet	2017 Aug 24	4		
7	Vientiane	Meungsong	Trade	Dry	2016 Mar 17	4	11 (3.1)	2 (18.2)
				Wet	2015 Aug 9	5		
				Wet	2017 Aug 25	2		
8	Vientiane	Pha-hom	Trade (roadside market)	Wet	2016 Jun 15	1	1 (0.3)	0/1 (0.0)
9	Xiengkhuang	Nam-ngam	Trade	Wet	2016 May 12	4	14 (3.9)	3 (21.4)
				Wet	2016 May 13	3		
				Wet	2016 May 16	6		
				Wet	2017 Aug 22	1		
10	Xiengkhuang	Phonsavanh/ Souanphukham	Trade	Dry	2014 Dec 18	2	34 (9.5)	6 (17.6)
				Dry	2016 Mar 11	9		
				Dry	2016 Mar 12	5		
				Dry	2016 Mar 13	5		
				Wet	2015 Sep 15	4		
				Wet	2015 Sep 16	4		
				Wet	2015 Sep 17	4		
				Wet	2016 May 15	1		
11	Xiengkhuang	Thajok	Trade (roadside stall)	Wet	2016 May 13	3	4 (1.1)	1 (25.0)
				Wet	2016 May 16	1		
BKX	Bolikhamxay	Bolikhamxay POFI	POFI	Dry	2014 Nov 13	4	28 (7.8)	22 (78.6)
				Dry	2015 Apr 23	4		
				Dry	2017 Jan 18	6		
				Wet	2014 Jul 10	2		
				Wet	2016 Jul 26	5		
				Wet	2016 Aug 29	5		
				Wet	2016 Aug 30	2		
CHP	Champasak	Champasak POFI	POFI	Wet	2017 May 19	1	1 (0.3)	1 (100.0)
VTE	Vientiane Capital	Vientiane Capital POFI	POFI	Wet	2016 Jul 7	6	6 (1.7)	2 (33.3)
TOTAL							359 (100.0)	92 (25.6)

*POFI, Provincial Office of Forestry Inspection.

Appendix Table 2. Sampling methods used in wildlife traded in markets for human consumption, Laos*

Sample type	Animal status	Method	Sample preservation
Urogenital swab	Live or dead	Urogenital area swabbed (Puritan Medical Products, https://www.puritanmedproducts.com), collected in duplicate.	VTM + RNAlater†
Urine	Live	Plastic sheet was placed under cages and left until the animal urinated (or for a maximum of 30 min). Collected urine was transferred to tube.	Plain tube
	Dead	Collected either by using a disposable pipette after pressing on the bladder or by cystocentesis using a 21G needle and sterile syringe. When insufficient urine was available, the urogenital area was swabbed up to 2 times.	Plain tube *or* VTM + RNAlater (swabs)
Blood	Dead	Blood drawn by rib cage cardiac puncture using a 21G needle	Plain tube
Liver	Dead (nonbutchered)	Obtained using a punch biopsy needle (Single Action Biopsy Device 14G, 20 mm Throw Trocar Tip, Argon Medical Devices, https://www.argonmedical.com).	VTM + RNAlater
Liver, kidney, or spleen	Dead (butchered or collected by POFI)	≈200 mg tissue samples collected	VTM + RNAlater

*POFI, Provincial Office of Forestry Inspection; VTM, viral transport medium (in-house formulation, National Animal Health Laboratories, Vientiane, Laos).
†RNAlater (Sigma-Aldrich, https://www.sigmaaldrich.com).

Appendix Table 3. PCR assays used for pathogen detection in wildlife traded in markets for human consumption, Laos*

Pathogen	Name	Sequence 5′-3′	Target region	Reference
O. tsutsugamushi	OtsuFP630	AACTGATTTTATTCAAACTAATGCTGCT	47 kDa outer membrane protein	(*4*)
	OtsuRP747	TATGCCTGAGTAAGATACRTGAATRGAATT		
	OtsuPR665	FAM-TGGGTAGCTTTGGTGGACCGATGTTTAATCT-TAMRA		
Rickettsia spp.	R17K128F2	GGGCGGTATGAAYAAACAAG	17 kDa surface antigen	(*4*)
	R17K238R	CCTACACCTACTCCVACAAG		
	R17K202TAQP	FAM-CCGAATTGAGAACCAAGTAATGC-TAMRA		
Nested *Rickettsia* spp.	R17kM61F	ACTTTACAAAATTCTAAAAACCATATACT	17 kDa surface antigen	(*5*)
	R17K31F	GCTCTTGCAGCTTCTATGTTACA		
	Rr2608Rnew	CATTGTCCGTCAGGTTGGCG		
Leptospira spp.	Lepto-F	CCCGCGTCCGATTAG	16s rRNA	(*6–8*)
	Lepto-R	TCCATTGTGGCCGRACAC		
	Lepto-probe	FAM-CTCACCAAGGCGACGATCGGTAGC-BHQ1		
Anaplasmataceae (*Neorickettsia* spp./*Anaplasma* spp./*Ehrlichia* spp.)	Ehr-16S_F	GGTACCYACAGAAGAAGTCC	16s rRNA	(*9*)
	Ehr-16S_R	TAGCACTCATCGTTTACAGC		
E. chaffeensis	ECH16S-17	GCGGCAAGCCTAACACAT	16s rRNA	(*10*)
	ECH16S-97	CCCGTCTGCCACTAACAATTATT		
	ECH16S-38	carboxyfluorescein-AGTCGAACGGACAATTGCTTATAACCTTTTGGT		
A. phagocytophilum	ApMSP2f	ATCGAAGGTAGTGTTGGTTATGGTATT	msp2 outer membrane protein	(*11*)
	APMSP2r	TTGGTCTTGAAGCGCTCGTA		
	APMAP2p	HEX-TGGTGCCAGGGTTGAGCTTGAGATTG-TAMRA		
C. burnetii	IS1111f	CAAGAAACGTATCGCTGTGGC	IS1111 transposase	(*12*)
	IS1111R	CACAGAGCCACCGTATGAATC		
	IS1111 probe	FAM-CCGAGTTCGAAACAATGAGGGCTG-TAMRA		
Flavivirus	PF1	TGYRTBTAYAACATGATGGG	NS5	(*13*)
	PF2bis	GTGTCCCAICCNGCNGTRTC		
	PF3	ATHTGGTWYATGTGGYTDGG		
Dengue virus	DenAll-F	AGGACYAGAGGTTAGAGGAGA	3′UTR	(*14*)
	DenAll-R	CGYCTCTGTGCCTGGAWTGAT		
	DenAll-P	FAM-ACAGCATATTGACGCTGGGARAGACC-TAMRA		
Hantavirus	PanHanta-F2	TGCWGATGCIACRAAATGGTC	L segments	(*15*)
	PanHanta-R2	GCATCATCWGARTGATGIGCAA		
Zika virus	ZIKA2 S	CTTGGAGTGCTTGTGATT	Polyprotein	†
	ZIKA2 R	CTCCTCCAGTGTTCATTT		
	ZIKA2 PROBE	FAM-AGAAGAGAATGACCACAAAGATCA-TAMRA		
Universal	27F/V1-F	AGAGTTTGATCMTGGCTCAG	16S rRNA	(*16, 17*)
	518R/V3-R	GTATTACCGCGGCTGCTGGCA		
T4	T4F	CCATCCATAGAGAAAATATCAGAACGA	Enterobacteria phage T4	(*18*)
	T4R	TAAATAATTCCTCTTTTCCCAGCG		
	T4probe	VIC-AACCAGTAATTTCATCTGCTTCTGATGTGAGGC-TAMRA		
MS2	MS2F	CTCTGAGAGCGGCTCTATTGGT	Enterobacteria phage MS2	(*18*)
	MS2R	GTTCCCTACAACGAGCCTAAATTC		
	MS2probe	VIC-TCAGACACGCGGTCCGCTATAACGA-TAMRA		

*Assays used 5 µL of DNA/RNA template with a 20 µL PCR mastermix containing 0.8 µg/µL BSA. Bacterial quantitative PCRs used Platinum Quantitative PCR SuperMix-UDG (ThermoFisher Scientific, https://www.thermofisher.com), while either SuperScript III Platinum One-Step qRT-PCR system (ThermoFisher Scientific) or QuantiTect SybrGreen quantitative reverse transcription PCR kit (QIAGEN, https://www.giagen.com) was used for viral quantitative reverse transcription PCR. A cycle threshold value of <40 was classified as positive. Where applicable, primers were also used for sequencing of PCR products.

†Inhouse quantitative reverse transcription PCR by Unité des Virus Emergents, L'Institut de recherche pour le développement, Faculté de Médecine, Timone, Marseille, France.

Appendix Table 4. Number and type of animal species sampled in wildlife markets, Laos

Order	Family	Scientific name	Common name	No. (%)
Artiodactyla	Cervidae	*Muntiacus muntjak*	Red muntjac	1 (0.3)
Carnivora	Felidae	*Catopuma temminckii*	Asian golden cat	1 (0.3)
		Prionailurus bengalensis	Leopard cat	3 (0.8)
	Herpestidae	*Herpestes javanicus*	Small Asian mongoose	3 (0.8)
	Mustelidae	*Martes flavigula*	Yellow-throated marten	1 (0.3)
	Viverridae	*Arctogalidia trivirgata*	Small-toothed palm civet	2 (0.6)
		Paguma larvata	Masked palm civet	2 (0.6)
		Paradoxurus hermaphroditus	Common palm civet	24 (6.7)
		Viverra megaspila	Large-spotted civet	1 (0.3)
		Viverricula indica	Small Indian civet	1 (0.3)
Chiroptera	Pteropodidae	*Cynopterus* sp.	Bat sp.	3 (0.8)
		Eonycteris spelaea	Cave nectar bat	3 (0.8)
		Macroglossus sobrinus	Greater Long-tongued nectar bat	1 (0.3)
		Megaerops sp.	Bat sp.	2 (0.6)
		Rousettus sp.	Bat sp.	14 (3.9)
		Sphaerius sp.	Bat sp.	2 (0.6)
Lagomorpha	Leporidae	*Lepus peguensis*	Burmese hare	1 (0.3)
Rodentia	Hystricidae	*Atherurus macrourus*	Brush-tailed porcupine	1 (0.3)
	Muridae	Unknown	Rat sp.	1 (0.3)
	Sciuridae	*Belomys pearsonii*	Hairy-footed flying squirrel	12 (3.3)
		Callosciurus erythraeus	Pallas's squirrel	73 (20.3)
		Callosciurus finlaysonii	Variable squirrel	29 (8.1)
		Callosciurus inornatus	Inornate squirrel	40 (11.1)
		Dremomys rufigenis	Red-cheeked squirrel	36 (10.0)
		Hylopetes alboniger	Particolored flying squirrel	5 (1.4)
		Hylopetes phayrei	Phayre's flying squirrel	9 (2.5)
		Hylopetes sp.	Small flying squirrel	4 (1.1)
		Hylopetes spadiceus	Red-cheeked flying squirrel	2 (0.6)
		Menetes berdmorei	Indochinese ground squirrel	29 (8.1)
		Petaurista elegans	Lesser giant flying squirrel	1 (0.3)
		Petaurista petaurista	Red giant flying squirrel	2 (0.6)
		Petaurista philippensis	Indian giant flying squirrel	14 (3.9)
		Petaurista sp.	Giant flying squirrel	1 (0.3)
		Ratufa bicolor	Black giant squirrel	3 (0.8)
		Unknown	Small flying squirrel	2 (0.6)
	Spalacidae	*Rhizomys pruinosus*	Hoary bamboo rat	21 (5.8)
		Rhizomys sumatrensis	Indomalayan bamboo rat	6 (1.7)
Scandentia	Tupaiidae	*Tupaia belangeri*	Northern treeshrew	3 (0.8)
Total				359

Appendix Table 5. Wildlife specimens collected in markets and results of PCR tests for zoonotic pathogens, Laos*

		No. positive/No. tested											
Animal specimens from trade sites	No.	16S Universal cPCR	*Leptospira* spp. q-PCR	*Coxiella* q-PCR	*Anaplasma phagocytophilum* q-PCR	*Ehrlichia chaffeensis* q-PCR	*Orientia tsutsugamushi* q-PCR	Anaplasmatacae cPCR	*Rickettsia* spp. qPCR	*Rickettsia typhi* qPCR	Dengue virus qRT-PCR	Flavivirus qRT-PCR	Zika virus qRT-PCR
Arctogalidia trivirgata	2	–	2/2	n.s	n.s	n.s	n.s	n.s	n.s	–	0/2	0/2	0/2
Atherurus macrourus	1	–	1/1	n.s	n.s	n.s	n.s	n.s	n.s	–	0/1	0/1	0/1
Belomys pearsonii	12	–	1/12	n.s	n.s	n.s	n.s	n.s	n.s	–	0/12	0/12	0/12
Callosciurus erythraeus	56	–	8/56	0/9	0/9	0/9	0/9	0/9	0/8	–	0/56	0/56	0/55
Callosciurus finlaysonii	28	–	13/28	0/2	0/2	0/2	0/2	0/2	0/2	–	0/28	0/28	0/28
Callosciurus inornatus	34	–	7/34	0/3	0/3	0/3	0/3	0/3	0/3	–	0/34	0/34	0/34
Cynopterus spp.	3	–	0/3	n.s	n.s	n.s	n.s	n.s	n.s	–	0/3	0/3	0/3
Dremomys rufigenis	35	–	5/35	0/11	0/11	0/11	0/11	0/11	2/11	0/1	0/35	0/35	0/35
Eonycteris spelaea	3	–	1/3	0/2	0/2	0/2	0/2	0/2	0/2	–	0/3	0/3	0/3
Herpestes javanicus	3	–	0/3	0/2	0/2	0/2	0/2	0/2	0/2	–	0/3	0/3	0/3
Hylopetes alboniger	5	–	1/5	n.s	n.s	n.s	n.s	n.s	n.s	–	0/5	0/5	0/5
Hylopetes phayrei	9	–	1/9	n.s	n.s	n.s	n.s	n.s	n.s	–	0/9	0/9	0/9
Hylopetes spp.	4	–	0/4	n.s	n.s	n.s	n.s	n.s	n.s	–	0/4	0/4	0/4
Hylopetes spadiceus	2	–	1/2	n.s	n.s	n.s	n.s	n.s	n.s	–	0/2	0/2	0/2
Lepus peguensis	1	–	0/1	n.s	n.s	n.s	n.s	n.s	n.s	–	0/1	0/1	0/1
Macroglossus sobrinus	1	–	0/1	n.s	n.s	n.s	n.s	n.s	n.s	–	0/1	0/1	0/1
Martes flavigula	1	–	0/1	0/1	0/1	0/1	0/1	0/1	0/1	–	0/1	0/1	0/1
Megaerops spp.	2	–	0/2	n.s	n.s	n.s	n.s	n.s	n.s	–	0/2	0/2	0/2
Menetes berdmorei	29	–	4/29	0/1	0/1	0/1	0/1	0/1	0/1	–	0/29	0/29	0/29
Muntiacus muntjak	1	–	1/1	0/1	0/1	0/1	0/1	1/1	0/1	–	0/1	0/1	0/1
Paguma larvata	2	–	1/2	n.s	n.s	n.s	n.s	n.s	n.s	–	0/2	0/2	0/2
Paradoxurus hermaphroditus	22	–	10/22	0/6	0/6	0/6	0/6	1/6	1/6	0/1	0/22	0/22	0/22
Petaurista elegans	1	–	0/1	n.s	n.s	n.s	n.s	n.s	n.s	–	0/1	0/1	0/1
Petaurista petaurista	2	–	0/2	n.s	n.s	n.s	n.s	n.s	n.s	–	0/2	0/2	0/2
Petaurista philippensis	9	–	1/9	0/2	0/2	0/2	0/2	0/2	1/2	–	0/9	0/9	0/9
Petaurista spp.	1	–	0/1	0/1	0/1	0/1	0/1	0/1	0/1	–	0/1	0/1	0/1
Prionailurus bengalensis	3	–	1/3	n.s	n.s	n.s	n.s	n.s	n.s	–	0/3	0/3	0/3
Ratufa bicolor	2	–	0/2	n.s	n.s	n.s	n.s	n.s	n.s	–	0/2	0/2	0/2
Rhizomys pruinosus	21	–	3/21	n.s	n.s	n.s	n.s	n.s	n.s	–	0/21	0/21	0/21
Rhizomys sumatrensis	6	–	1/6	n.s	n.s	n.s	n.s	n.s	n.s	–	0/6	0/6	0/6
Rousettus spp.	14	–	0/14	n.s	n.s	n.s	n.s	n.s	n.s	–	0/14	0/14	0/13
Sphaerius spp.	2	–	0/2	n.s	n.s	n.s	n.s	n.s	n.s	–	0/2	0/2	0/2
Tupaia belangeri	3	–	1/3	n.s	n.s	n.s	n.s	n.s	n.s	–	0/3	0/3	0/3
Unknown Sciuridae	2	–	1/2	n.s	n.s	n.s	n.s	n.s	n.s	–	0/2	0/2	0/2
Viverra megaspila	1	–	0/1	n.s	n.s	n.s	n.s	n.s	n.s	–	0/1	0/1	0/1
Viverricula indica	1	–	0/1	n.s	n.s	n.s	n.s	n.s	n.s	–	0/1	0/1	0/1
Animal specimens from POFI													
Callosciurus erythraeus	17	2/2	4/17	0/17	0/17	0/17	0/17	0/17	11/17	6/11	0/17	0/17	0/16
Callosciurus finlaysonii	1	–	1/1	0/1	0/1	0/1	0/1	0/1	0/1	–	0/1	0/1	0/1
Callosciurus inornatus	6	–	2/6	0/6	0/6	0/6	0/6	0/6	2/6	0/2	0/6	0/6	0/6
Catopuma temminckii	1	–	1/1	0/1	0/1	0/1	0/1	0/1	1/1	0/1	0/1	0/1	0/1
Dremomys rufigenis	1	–	0/1	0/1	0/1	0/1	0/1	0/1	0/1	–	0/1	0/1	0/1
Paradoxurus hermaphroditus	2	–	0/2	0/2	0/2	0/2	0/2	0/2	1/2	–	0/2	0/2	0/2
Petaurista philippensis	5	–	1/5	0/4	0/4	0/4	0/4	0/4	3/5	0/2	0/5	0/5	0/5
Ratufa bicolor	1	–	0/1	0/1	0/1	0/1	0/1	0/1	1/1	0/1	0/1	0/1	0/1

Animal specimens from trade sites	No.	No. positive/No. tested											
		16S Universal cPCR	*Leptospira* spp. q-PCR	*Coxiella* q-PCR	*Anaplasma phagocytophilum* q-PCR	*Ehrlichia chaffeensis* q-PCR	*Orientia tsutsugamushi* q-PCR	Anaplasmatacae cPCR	*Rickettsia* spp. qPCR	*Rickettsia typhi* qPCR	Dengue virus qRT-PCR	Flavivirus qRT-PCR	Zika virus qRT-PCR
Unknown Muridae	1	–	0/1	0/1	0/1	0/1	0/1	0/1	0/1	–	0/1	0/1	0/1
Sample types taken from trade sites													
Blood	85	–	9/85	0/1	0/1	0/1	0/1	0/1	0/1	–	0/85	0/85	0/85
Kidney	6	–	2/6	0/6	0/6	0/6	0/6	4/6	0/6	–	0/6	0/6	0/6
Liver	40	–	1/40	0/40	0/40	0/40	0/40	3/40	0/40	0/2	0/40	0/40	0/40
Spleen	3	–	1/3	0/3	0/3	0/3	0/3	1/3	0/3	–	0/3	0/3	0/3
Urine	15	–	1/15	n.s	n.s	n.s	n.s	n.s	n.s	–	0/15	0/15	0/14
Urogenital swab	312	–	58/312	0/18	0/18	0/18	0/18	0/18	0/18	–	0/312	0/312	0/311
Sample types from POFI-collected animals													
Blood	3	–	1/3	n.s	n.s	n.s	n.s	n.s	n.s	–	0/3	0/3	0/3
Kidney	91	–	14/91	0/91	0/91	0/91	0/91	5/91	29/91	4/27	0/91	0/91	0/90
Liver	92	1/1	14/92	0/92	0/92	0/92	0/92	4/92	34/92	2/29	0/92	0/92	0/92
Spleen	69	1/1	17/69	0/69	0/69	0/69	2/69	8/69	17/69	1/17	0/69	0/69	0/69
Urogenital swab	1	–	0/1	n.s	n.s	n.s	n.s	n.s	n.s	–	0/1	0/1	0/1

*cPCR, conventional PCR; n.s., no appropriate sample available; POFI, Provincial Office of Forestry Inspection; qPCR, quantitative PCR; qRT-PCR, quantitative reverse transcription PCR; –, not tested;

Appendix Table 6. Descriptions of organisms identified in wildlife traded for human consumption, Laos

Organism	Human pathogen	Notes	Reference
Rickettsia typhi	Yes	Cause of murine typhus, a major underrecognized cause of fever in Laos	(*19*)
Rickettsia felis	Yes	An emerging rickettsial pathogen, often misdiagnosed as other febrile illnesses	(*20*)
Orientia tsutsugamushi	Yes	Cause of scrub typhus, responsible for up to 23% of fever cases in Laos. Vectors are *Leptotrombidium* mites. Commonly associated with ground-dwelling rodents, but vectors are known to parasitize squirrels. *O. tsutsugamushi* has been isolated from *Callosciurus notatus* (plantain squirrel) in Malaysia	(*21–23*)
Anaplasma phagocytophilum	Yes	Cause of human granulocytic anaplasmosis	(*24*)
Anaplasma platys	Yes	Been identified in those with close associations with infected animals, such as veterinarians and companion animal owners	(*25*)
Anaplasma capra	Yes	Has been identified in humans following tick bites	(*26*)
Anaplasma marginale	No	Closely related to *A. centrale*. Known cause of bovine anaplasmosis	(*27*)
Anaplasma centrale	No	A known cause of bovine anaplasmosis	(*27*)
Anaplasma bovis	No	A known cause of bovine anaplasmosis. Not reported to infect humans	(*24*,*28*,*29*)
Ehrlichia chaffeensis	Yes	Cause of human monocytic ehrlichiosis	(*30*)
Lactococcus garvieae	Rare	Rare cause of human opportunistic infections	(*31*)
Kurthia species	Rare	Rare cause of human opportunistic infections	(*32*)
